# Supplementary material for: The Anti-Cancer Activity of Pentamidine and Its Derivatives (WLC-4059) Is through Blocking the Interaction between S100A1 and RAGE V Domain
Source: Biomolecules. 2022 Dec 30;13(1):81. doi: 10.3390/biom13010081 (PMC9856166; doi:10.3390/biom13010081)
Supplement: Supplementary file 1 [file biomolecules-13-00081-s001.zip › biomolecules-1973296-supplementary.pdf]

Figure S1. HPLC, SDS PAGE and ESI mass of S100A1.

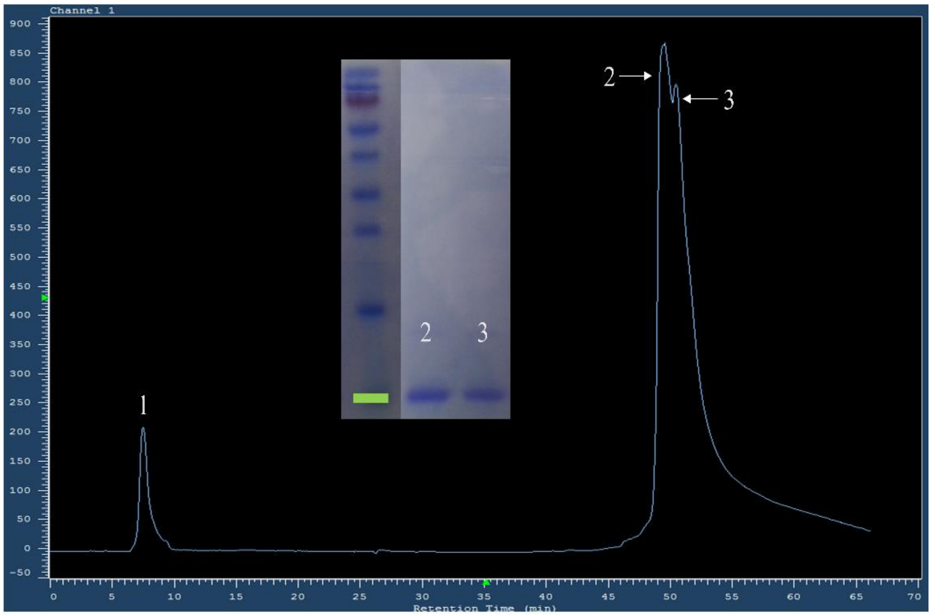

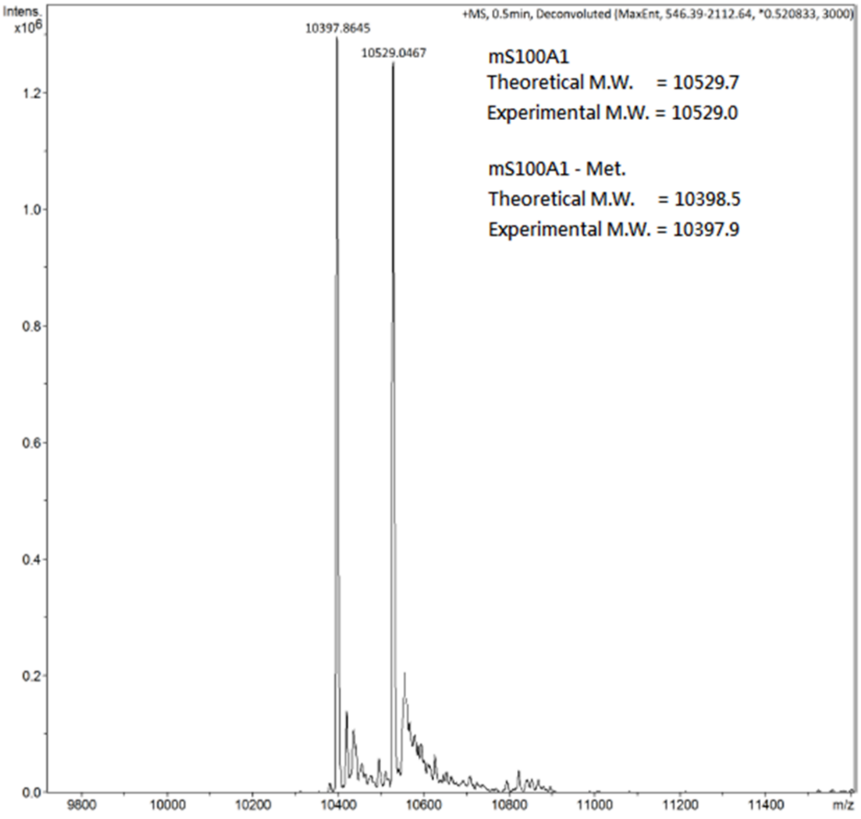

Figure S2: NMR data of the pentamidine derivative WLC-4059.

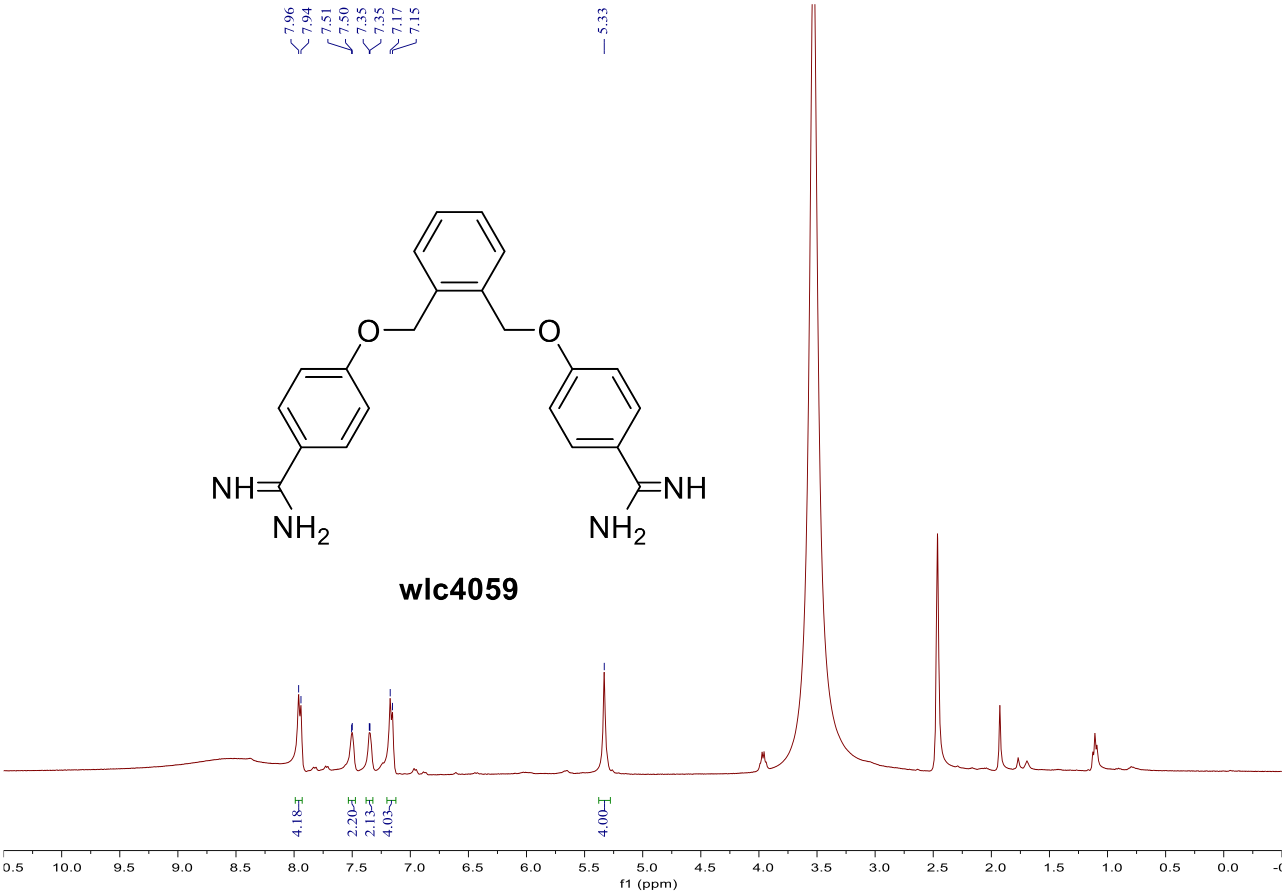

^{1}H NMR (400 MHz, DMSO-d_6) spectrum of **wlc4059**

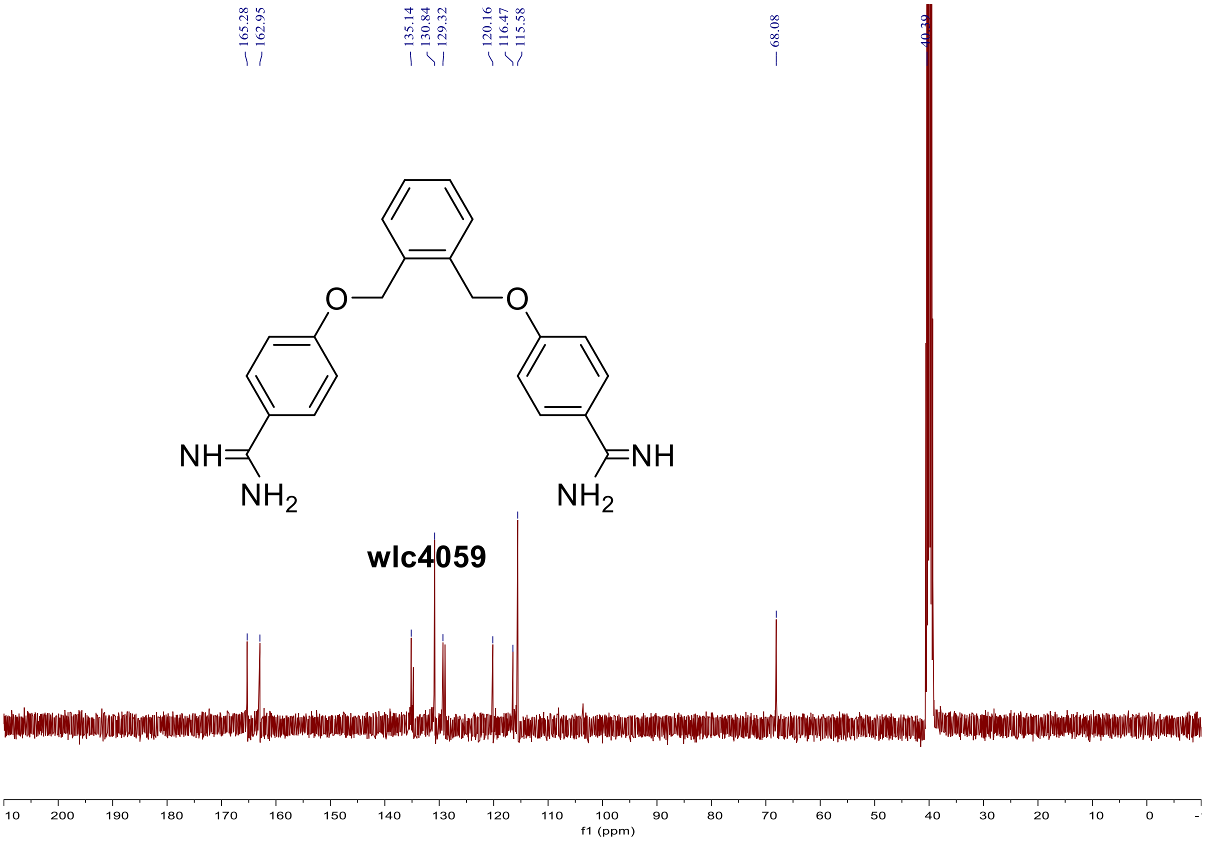

^{13}C NMR (400 MHz, DMSO-d^6) spectrum of **wlc4059**

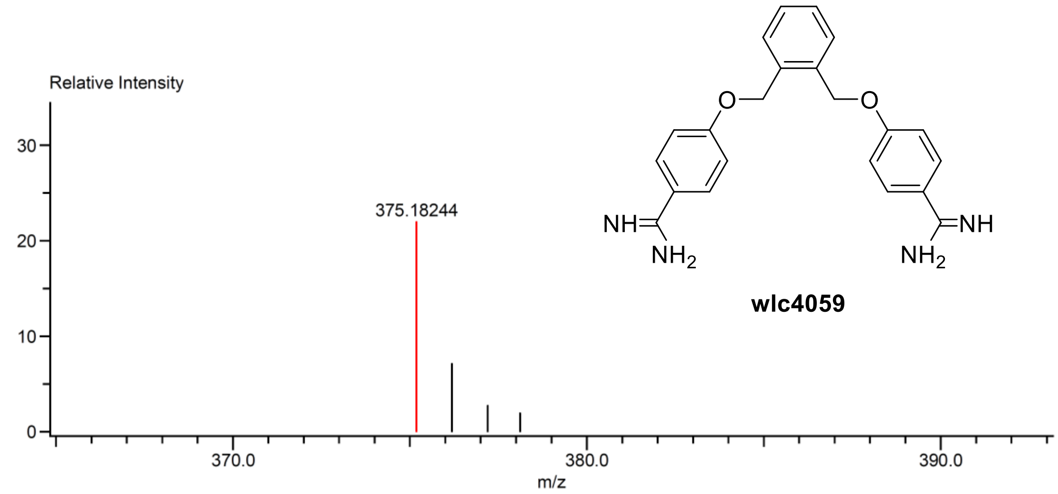

HRMS of compound **wlc4059**

Figure S3. The clinical corelation of RAGE in patients with colorectal cancer

A B

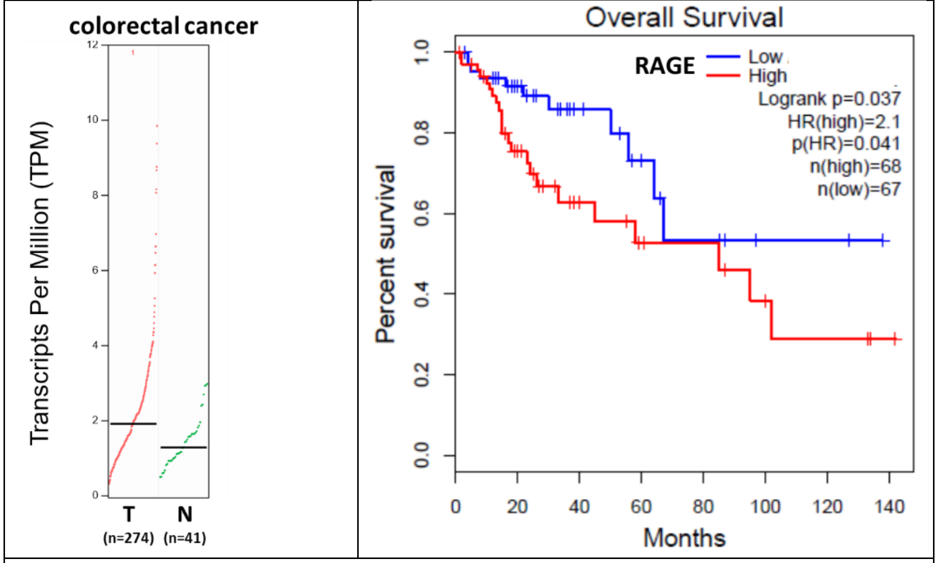

Figure S3. The clinical corelation of RAGE in pateins with colorectal cancer. The gene expression and clinical data were obtained and analyzed from GEPIA database. (A) The expression of RAGE in tumorius (T) and normal (N) tissues from the pateins with colorectal cancer. (B) Overall survival of patients with high (red line) and low (blue line) levels of RAGE.
